# Effects of High Hydrostatic Pressure Pretreatment on the Functional and Structural Properties of Rice Bran Protein Hydrolysates

**DOI:** 10.3390/foods11010029

**Published:** 2021-12-23

**Authors:** Shirang Wang, Tengyu Wang, Yue Sun, Yingju Cui, Guoping Yu, Lianzhou Jiang

**Affiliations:** 1 College of Food Science, Northeast Agricultural University, Harbin 150030, China; wangshirang2017@126.com (S.W.); 15533765253@163.com (Y.S.); cyingjy@163.com (Y.C.); jlzname@163.com (L.J.); 2 School of Grain Engineering, Heilongjiang Communications Polytechnic, Harbin 150025, China; wangtengyu2017@126.com

**Keywords:** high hydrostatic pressure, trypsin, hydrolysis, rice bran protein hydrolysate, solubility, emulsifying property, protein structure

## Abstract

Rice bran protein (RBP) hydrolysis was conducted after high hydrostatic pressure (HHP) pretreatment. The structural and functional properties of HHP-pretreated rice bran protein hydrolysates (RBPH) were investigated. HHP pretreatments were conducted at 100, 200, and 300 MPa; then, enzymatic hydrolysis at atmospheric pressure was performed using trypsin. An RBPH sample that had not been pretreated by HHP was used as a control. Free sulfhydryl (SH) content, SDS-PAGE profiles, high-performance size exclusion chromatography (HPSEC), Fourier transform infrared (FTIR) spectrum, scanning electron microscopy (SEM), intrinsic fluorescence spectrum, solubility, and emulsifying and foaming properties were evaluated. Changes in particle size and ζ-potential were monitored. Compared with the control, the results of solubility, the emulsifying activity index (EAI) and the emulsifying stability index (ESI) increased significantly (*p* < 0.05) at 200 MPa. The content of free SH increased significantly (*p* < 0.05) at 100 MPa. FTIR spectrum and fluorescence analysis confirmed the changes in the secondary and tertiary structures. The experimental results indicated that the structural and functional properties of HHP-pretreated RBPH improved.

## 1. Introduction

Rice bran is a low-priced, underutilized major by-product of rice processing [1]. Rice bran contains protein (12–16%), fat (15–20%), and fiber (23–28%) [2]. It also contains many other nutrients, such as phytosterols, vitamins, and antioxidants [3]. Rice bran protein (RBP), which contains lysine (3–4%), is a high-quality resource of protein for the food processing industry, and the content of lysine in RBP is much higher than that of proteins from many other cereal brans or legumes [4,5]. The protein efficiency ratio of RBP (1.6–1.9) is comparable with that of casein (2.5) [5]. RBP is used in the modern infant food industry also because it is a low-allergy protein [6,7]. The demand for cheap and high-quality plant proteins for applications in food processing, especially for infant formula, is increasing, and scholars have been searching for this kind of protein for a few years [8,9]. Low solubility limits the application of natural RBP owing to the disulfide bonds and hydrophobic interactions in RBP, which control the spatial structure of protein [10,11]. Consequently, proper methods of modification are required to obtain suitable functional and structural protein properties.

Physical, chemical, and enzymatic modification are common modification methods used at present [12]. High hydrostatic pressure (HHP) processing is a new technology which could improve food quality and shelf life, and it may be a suitable processing method for the food industry [13,14,15]. As reports have shown, HHP treatment could change the tertiary and quaternary structures of proteins. This technology has already been applied to reduce or eliminate the allergenicity of protein, and improve the sensory and nutritional characteristics of food processing ingredients while inactivating microorganisms [16,17]. Some scholars have studied the functional and nutritional properties of soy protein isolate [18,19] and RBP [20] only processed by HHP treatment. Wang et al. [18] investigated the effects of 200–600 MPa high-pressure treatment on some physicochemical and functional properties of soy protein isolates at different concentrations. The study indicated that proper protein concentration and suitable HHP treatment could affect the emulsifying activity, solubility, and gelling property of soybean protein. Li et al. [19] studied the effects of HHP on the functional and nutritional characteristics of soy protein isolate in infant formula. The paper found that soy protein treated by HHP also showed better in vitro digestibility. Zhu et al. [20] reported that HHP treatment under 100–500 MPa pressure could modify functional properties such as gelling property, emulsifying property, emulsion stability etc. of RBP. This research also showed the relationship between surface hydrophobicity and the functional properties. Enzymatic modification is well known as a safe modification method to expose and release bioactive peptides without leading to protein nutrient loss [21]. Many scholars have carried out research into enzymatic protein modification, such as using rice bran, whey, corn, etc. as raw materials. Through enzymatic protein modification, the emulsification, freeze–thaw stability and oxidation resistance of protein have been improved [22,23,24,25,26]. HHP treatment is applied not only in novel food research but also in combination with enzymatic hydrolysis treatment to improve the functional properties of proteins [9]. Some scholars have reported the protease hydrolysis of soy protein isolate and lentil protein during HHP treatment [9,27]. Other investigators have studied the hydrolysis of soybean whey protein and ginkgo seed protein after HHP pretreatment [28,29]. Literature about the effects of HHP pretreatment on the structural and functional properties of rice bran protein hydrolysates (RBPH) have largely been unexplored.

The objective of the current work was to investigate the influence of HHP pretreatment on the structural and functional properties of RBPH, including its solubility, emulsifying activity index (EAI), emulsifying stability index (ESI), foaming capacity (FC), and foaming stability (FS). Besides, the mechanism of HHP pretreatment on RBPH was studied by observing the particle size and ζ-potential, sodium dodecyl sulfate-polyacrylamide gel electrophoresis (SDS-PAGE), scanning electron microscopy (SEM), high-performance size exclusion chromatography (HPSEC) and Fourier transform infrared (FTIR) spectrum. The study will provide a basis for further elucidating the mechanisms of the functional properties of RBP modified by HHP pretreatment.

## 2. Materials and Methods

### 2.1. Materials

Fresh rice bran was purchased from Heilongjiang Great Northern Wilderness Agribusiness Group Co., Ltd. (Harbin, Heilongjiang, China). RBP was obtained in laboratory condition. Trypsin (3 × 10^4^ U/g) was obtained from Sigma-Aldrich Co. (St. Louis, MO, USA). Deionized water was used. Chemicals and reagents were of analytical grade. 

### 2.2. Extraction of RBP

RBP was extracted in the laboratory according to Zang et al. [24]. Rice bran was defatted for 4 h with 10 volumes of N-hexane by magnetic stirring. The defatted rice bran was air-dried for 24 h at 25 °C. Tenfold deionized water was added to the defatted rice bran by stirring for 1 h, then the pH of the solution was adjusted to 9.0 with 2 M NaOH. After being stirred for 2 h, the resulting suspension was centrifuged at 8000× *g* for 20 min at 4 °C. The pH of the supernatant was adjusted to 4.5 with 2 M HCl. The precipitated protein was obtained by centrifugation at 4000× *g* for 20 min at 4 °C. The protein was dispersed in fivefold deionized water and washed twice. After centrifugation at 6000× *g* for 10 min, the pH was adjusted to 7.0 with 2 M NaOH. The neutral RBP solution was freeze-dried in a freeze drier and stored at −20 °C. The protein content of this RBP was 91.67%, which was determined by the Kjeldahl method (N% × 5.95).

### 2.3. HHP Pretreatment

HHP pretreatment was carried out at 25 °C in HHP equipment (Ren-He Electromechanical Engineering CO., Shenyang, China), with water used as hydrostatic fluid. RBP was dispersed in a 0.01 M phosphate buffer (1%, *w*/*v*). RBP solutions (200 mL in each bag) were packed in polyethylene plastic bags, sealed after exhausting the air, placed in HHP equipment. Samples were treated by HHP at 100, 200, and 300 MPa for 30 min. The indicated pressure was reached in 1 to 2 min; this pressure was kept for 30 min and released to normal pressure in 1 to 2 min. Each HHP treatment was conducted three times.

### 2.4. Preparation of RBPH

The hydrolysis experiment was carried out after HHP treatment. Hydrolysis of RBP by trypsin was performed at atmospheric pressure (0.1 MPa) for 60 min according to Zang et al. [24], with some modifications. The RBP only hydrolyzed was used as a control. After the pH and temperature adjustments (pH = 8, 37 °C), trypsin was added (enzyme/substrate = 1/20 (*w*/*v*)). Hydrolysis was performed in triplicate, and the reaction was stopped by heating the hydrolysates for 10 min at 90 °C. Next, the pH of the solution was maintained at 7 with 0.5 M NaOH, and the supernatant was obtained by centrifugation at 6000× *g* for 15 min at 4 °C. The supernatant was then stored at −20 °C until analysis.

### 2.5. Solubility

Solubility was determined according to Zang et al. [24] with slight modifications. Briefly, the RBPH samples were diluted with a phosphate buffer (0.01 M, pH 7.0) to a protein concentration of 1% (*w*/*v*), and the pH was adjusted to 7.0. The dispersion was then centrifuged at 10,000× *g* for 20 min. The protein content in the supernatant was measured by Lowry’s method using bovine serum albumin (BSA) as the standard. Equation (1) was used to calculate the solubility.

(1) $$\mathrm{Protein}\;\mathrm{solubility}\;\left(\%\right)=\frac{\mathrm{Protein}\;\mathrm{content}\;\mathrm{in}\;\mathrm{the}\;\mathrm{supernatant}}{\mathrm{Total}\;\mathrm{protein}\;\mathrm{content}\;\mathrm{in}\;\mathrm{the}\;\mathrm{sample}}\;\times \;100$$

### 2.6. Emulsifying Properties

The procedures of Li et al. [30] were used to measure the emulsifying properties of RBPH. The RBPH samples were diluted with a phosphate buffer (0.01 M, pH 7.0) to a protein concentration of 0.5% (*w*/*v*), then 0.5% (*w*/*v*) of an RBPH (12 mL) solution was mixed with soybean oil (4 mL) to prepare the emulsion by homogenizing for 1 min at 10,000 rpm. Next, 50 μL of the microemulsion was removed from the bottom of the emulsion and immediately diluted with 5 mL of 0.1% sodium dodecyl sulfate (SDS) solution and vortexed. After that, the absorbance of the mixtures was measured at 500 nm for 0 min and 10 min; 0.5% (*w*/*v*) of the RBPH solution was used as a control. Equations (2) and (3) were used to calculate EAI and ESI, respectively:

(2) $$\mathrm{EAI}\left(m^{2}/g\right)=\frac{2\;\times \;2{.303\;\times \;A}_{0}\;\times \;100}{1000\;\times \;0.25\;\times \;1\;\times \;0.005}$$

(3) $$\mathrm{ESI}\left(\min\right)=\frac{A_{0}}{A_{0}\;{-\;A}_{10}}\;\times \;\left(T_{10\;}-{\;T}_{0}\right)$$

where A_0_ and A_10_ are the absorbance determined at 0 min and 10 min, respectively. T_0_ is 0 min, and T_10_ is 10 min.

### 2.7. Foaming Properties

FC and FS were measured by the method of Zhang et al. [31]. Briefly, the RBPH samples were diluted with a phosphate buffer (0.01 M, pH 7.0) to a protein concentration of 1% (*w*/*v*), and then 20 mL of 1% (*w*/*v*) of the RBPH sample solution diluted with deionized water was homogenized at 10,000 rpm for 1 min. The homogenized sample was left to stand for 30 min at room temperature to calculate the FS. FC and FS were calculated by Equations (4) and (5):

(4) $$\mathrm{FC}\left(\%\right)=\frac{V_{2}\;{-\;V}_{1}}{V_{1}}\;\times \;100$$

(5) $$\mathrm{FS}\left(\%\right)=\frac{V_{3}\;{-\;V}_{1}}{V_{2}}\;\times \;100$$

where V_1_ (mL) is volume before whipping, V_2_ (mL) is volume after whipping, V_3_ (mL) is volume after standing.

### 2.8. Particle Size

The samples were diluted with a phosphate buffer (0.01 M, pH 7.0) to a protein concentration of 1% (*w*/*v*). The particle size and polydispersity index (PDI) analyses were performed with a particle size distribution instrument (Nano ZS 90; Malvern Instrument Co., Ltd., Malvern, Worcestershire, UK). The particle size was characterized by the d4,3 (volume average particle size).

### 2.9. ζ-Potential Measurements

The samples were diluted with a phosphate buffer (0.01 M, pH 7.0) to a protein concentration of 1% (*w*/*v*). ζ-potential was determined by the methods of Tang et al. [32]. A Zetasizer Nano ZS 90 (Malvern Instrument Ltd., Malvern, Worcestershire, UK) was used to measure the ζ-potential of RBPH.

### 2.10. SDS-PAGE Analysis

SDS-PAGE analysis was done by the method of Laemmli [33]. The RBPH samples was mixed with electrophoretic sample buffer (10% glycerol, 0.5 M Tris-HCl buffer, 5% *β*- mercaptoethanol and 1% bromophenol blue; pH 6.8) and boiled in boiling water for 5 min. 6 μL solution of each sample was loaded into proper well separately after cooling to room temperature. The stacking gel and the separating gel experiments were carried out at 90 V and 120 V, respectively. The gels were stained by coomassie Brilliant Blue R-250.

### 2.11. Free Sulfhydryl (SH) Content Determination

SH content was determined by the method of Shimada & Cheftel [34], with minor modifications. The RBPH solution of 1% (*w*/*v*) was solubilized in 5 mL of a Tris-Gly buffer solution (0.086 M Tris, 0.09 M Gly, 0.004 M EDTA and 8 M urea; pH 8.0). This solution was added to a 2-nitrobenzoic acid (DTNB) (20 μL) reagent. The absorbance of the mixture was measured at 412 nm. The solution without DTNB was used as the blank.

The -SH content was calculated as Equation (6):

(6) $$-\mathrm{SH}\left(\mu \mathrm{mol}/g\right)=\frac{73{.53\;\times \;A}_{412}\;\times \;D}{C}$$

where A_412_ was absorbance at 412 nm, C was the solids content in protein solution (mg/mL), and D was the dilution factor. 

### 2.12. HPSEC Measurements

HPSEC experiments were carried out using an Agilent 1100 liquid chromatograph equipped with a photodiode array detector according to Guan et al. [27], with some modifications. A 0.1% (*w*/*v*) protein concentration was obtained with a phosphate buffer (0.01 M, pH 7.0). A 0.45 μm cellulose acetate membrane was used to remove impurities. The sample was injected onto a Shodex protein KW-804 column (Shodex Separation and HPLC Group, Tokyo, Japan), with a 0.01 M phosphate buffer (pH 7.0) and 0.3 M NaCl used as the mobile phase. The flow rate was set at 1 mL/min. The eluent was monitored at 280 nm.

### 2.13. Surface Morphology Analysis

The surface morphology of each sample was analyzed by Scanning electron microscopy (SEM, Hitachi SU8010, Tokyo, Japan). The samples were coated with Au using an ion sputter to a thickness of 15 nm and observed at an accelerating voltage of 5.0 kV.

### 2.14. Fourier Transform Infrared (FTIR) Spectroscopy Measurements

The structure of freeze-dried RBPH samples were measured by an FTIR spectrometer (PerkinElmer, Buckinghamshire, UK). The samples were pressed into pellets with KBr powder at a proportion of 1:100. The scanning range was set to 500–4000 cm^−1^ with a resolution of 4 cm^−1^ and 64 scans. 

### 2.15. Fluorescence Spectrometry Measurements

Fluorescence spectrometry measurements were carried out by excitation at 290 nm by the method of Liu et al. [35] with slight modifications. The RBPH samples were diluted with a phosphate buffer (0.01 M, pH 7.0) to a protein concentration of 1% (*w*/*v*). Emission wavelengths were collected between 300 and 450 nm with a constant slit of 2.5 nm.

### 2.16. Statistical Analysis

Statistical comparisons were made at the significance level of *p* < 0.05, and analysis of variance (ANOVA) and Duncan’s test were used. All experiments were conducted in triplicate and SPSS 22.0 software was used to express the results as the mean values ± standard deviations.

## 3. Results and Discussion

### 3.1. Effect of HHP Pretreatment on the Solubility of RBPH

**Figure 1:**
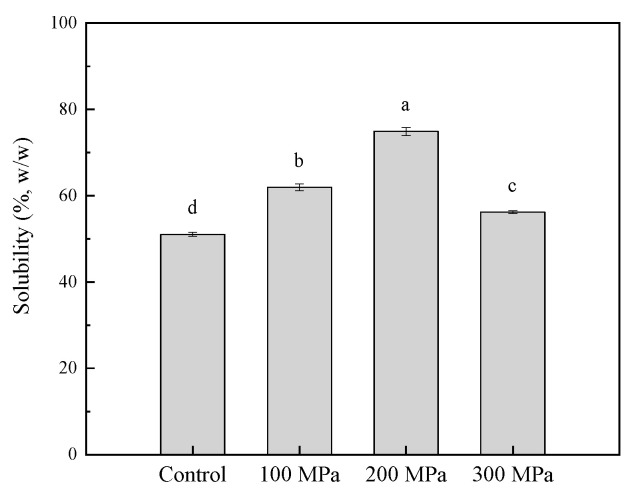
Effect of HHP pretreatment on the solubility of RBPH. HHP: high hydrostatic pressure; RBPH: rice bran protein hydrolysates. Control, 100 MPa, 200 MPa, and 300 MPa represent the RBPH samples subjected to 0, 100, 200, and 300 MPa pretreatment, respectively. Different lowercase letters indicate values that differ significantly (*p* < 0.05). All the HHP pretreatments were conducted at 25 °C for 30 min.

Solubility is crucial for protein in food applications [36]. Figure 1 shows the effect of HHP pretreatment on the solubility of RBPH. Compared with the control group that was not pretreated by HHP, the solubility of RBPH increased significantly (*p* < 0.05). This may be the reason why the structure of the protein became loose through the HHP pretreatment, especially the exposure of hydrophilic and hydrophobic groups. Enzyme action sites in protein that are easily enzymatically digested were exposed, and the exposure of hydrophilic groups can make protein disperse or hydrate more readily [20]. HHP pretreatment leads to better hydrolysis of RBP. Thus, the solubility improved greatly. When the pressure increased, the solubility increased gradually until 200 MPa, and then decreased. The decrease in solubility through HHP pretreatment beyond 200 MPa might be the reason for the buried hydrophobic groups being exposed when the protein was further unfolded [18]. 

### 3.2. Effect of HHP Pretreatment on the Emulsifying Properties of RBPH

**Figure 2:**
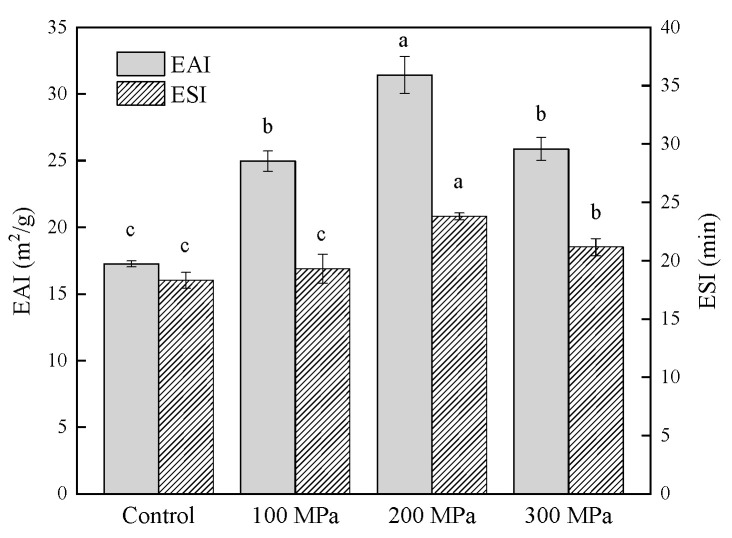
Effect of HHP pretreatment on the EAI and ESI of RBPH. HHP: high hydrostatic pressure; RBPH: rice bran protein hydrolysates; EAI: emulsifying activity index; ESI: emulsifying stability index. Control, 100 MPa, 200 MPa, and 300 MPa represent RBPH samples subjected to 0, 100, 200, and 300 MPa pretreatment, respectively. Different lowercase letters indicate values that differ significantly (*p* < 0.05). All the HHP pretreatments were conducted at 25 °C for 30 min.

The effects on the EAI and ESI of RBPH produced by HHP pretreatment are shown in Figure 2. Compared with the control group, EAI was significantly (*p* < 0.05) higher. When the pressure increased, EAI first increased and then decreased, and the maximal value was at 200 MPa. The present results share similarity to those of Wang et al. [18]. These results may be attributed to the unfolding of the RBP structure, which could increase RBP’s sorption at the oil–water interface, hence increasing the EAI [20]. HHP pretreatment increased the ESI of RBPH as well. From 100 to 200 MPa, the ESI of samples pretreated by HHP increased significantly (*p* < 0.05) compared with the ESI of the control group, whereas ESI decreased slightly at 300 MPa. The balance between hydrophilicity and lipophilicity determines the emulsifying properties of proteins [37]. The structure of RBP unfolded after the HHP pretreatment, which favored enzymatic hydrolysis. This phenomenon facilitated interactions between the protein and solvents to prevent the accumulation of oil droplets. Molecular flexibility has been proven to be an essential requisite for the stability of an emulsion [38]. The decrease in ESI at 300 MPa may be the reason why the molecular flexibility decreased under high pressure.

### 3.3. Effect of HHP Pretreatment on the Foaming Properties of RBPH

**Figure 3:**
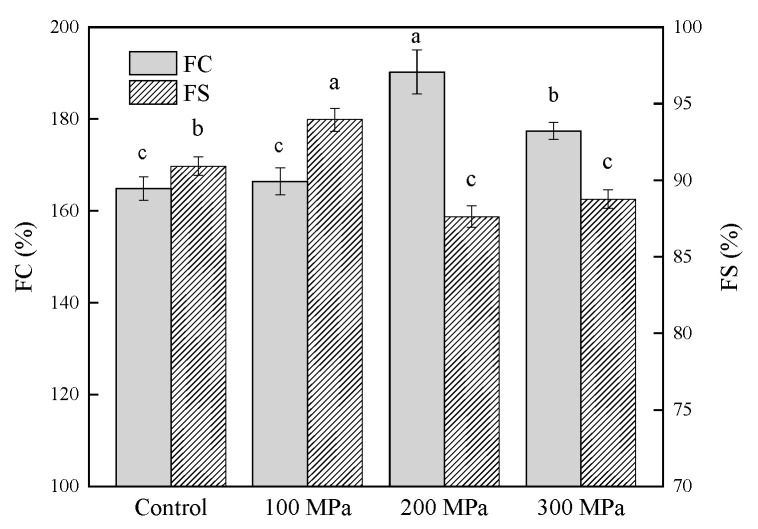
Effect of HHP pretreatment on the FC and FS of RBPH. HHP: high hydrostatic pressure; RBPH: rice bran protein hydrolysates; FC: foaming capacity; FS: foaming stability. Control, 100 MPa, 200 MPa, and 300 MPa represent RBPH samples subjected to 0, 100, 200, and 300 MPa pretreatment, respectively. Different lowercase letters indicate values that differ significantly (*p* < 0.05). All the HHP pretreatments were conducted at 25 °C for 30 min.

The FC and FS of RBPH pretreated by HHP are shown in Figure 3. The FC of HHP-pretreated RBPH showed a trend of first increasing and then decreasing with the increase in pressure, and the HHP pretreatment at 200 MPa produced the highest FC (*p* < 0.05). This trend is in parallel to the results of Maria et al. [39].

Compared with the control group, the FC of RBPH increased because of the HHP pretreatment, which increased the solubility of RBPH, unfolded part of the RBP structure, exposed buried hydrophobic groups, and increased the molecules’ flexibility, consequently promoting protein adsorption in the foaming process and reducing interfacial tension between air and water. Li et al. [19] stated that partially unfolded protein tended to form high viscoelastic and mechanical networks by means of a noncovalent interaction. The protein achieved good FC and FS, as the structure kept a suitable balance between flexibility and rigidity [40]. Therefore, the decrease in FC at 300 MPa may have been caused by the RBP structure’s rigidity and flexibility becoming unbalanced at this high level of pressure. The FS values at 100–300 MPa were 94%, 87.7%, and 88.7%, respectively, whereas that of the control group was 91%. The trend of these results was consistent with that of Zhu et al. [20].

In this study, the trend of the FC data was in accordance with the trends of solubility, EAI and ESI (Figure 1 and Figure 2).

### 3.4. Effect of HHP Pretreatment on the Particle Size of RBPH

**Figure 4:**
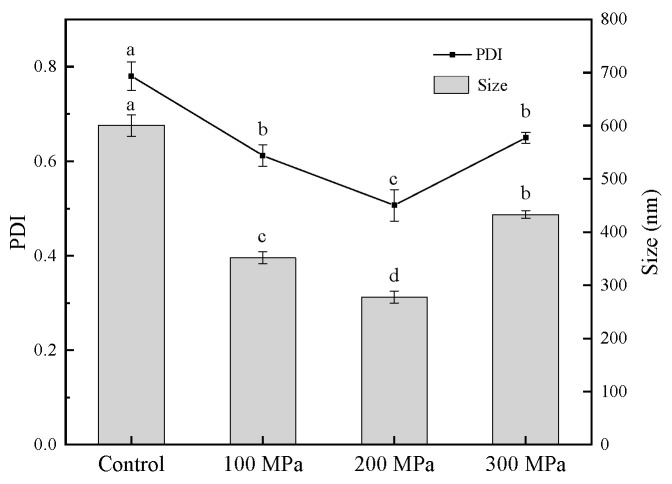
Effect of HHP pretreatment on the particle size and the PDI of RBPH. HHP: high hydrostatic pressure; RBPH: rice bran protein hydrolysates; PDI: polydispersity index; the particle size was characterized by the d4,3 (volume average particle size). Control, 100 MPa, 200 MPa, and 300 MPa represent RBPH samples subjected to 0, 100, 200, and 300 MPa pretreatment, respectively. Different lowercase letters indicate values that differ significantly (*p* < 0.05). All the HHP pretreatments were conducted at 25 °C for 30 min.

Figure 4 shows the volume average particle size (d4,3) and the PDI of the control and samples pretreated by HHP. As shown in the figure, the mean particle size tended to be significantly smaller (*p* < 0.05) compared with the control. PDI also exhibited the same tendency as particle size. As reported, high pressure made the particle size smaller [41]. The trend of these results was in line with that of the solubility index (Figure 1).

The data showed the average particle size of the control group was the largest out of all four samples. With the increase in pressure, the mean particle size of RBPH first decreased to 278 nm at 200 MPa, then increased to 433 nm at 300 MPa. The PDI showed the same trend. This result may be caused by the three-dimensional structure of RBP molecules becoming loose through HHP, as the RBP molecules had a certain degree of dissociation and extension [20]. The protein was hydrolyzed to a smaller particle size easily by trypsin. When the pressure was too high (300 MPa), the RBPH molecules aggregated, which may have been caused by the balance changes in RBPH’s spatial structure. Thus, the particle size became larger. 

### 3.5. Effect of HHP Pretreatment on the ζ-Potential of RBPH

**Figure 5:**
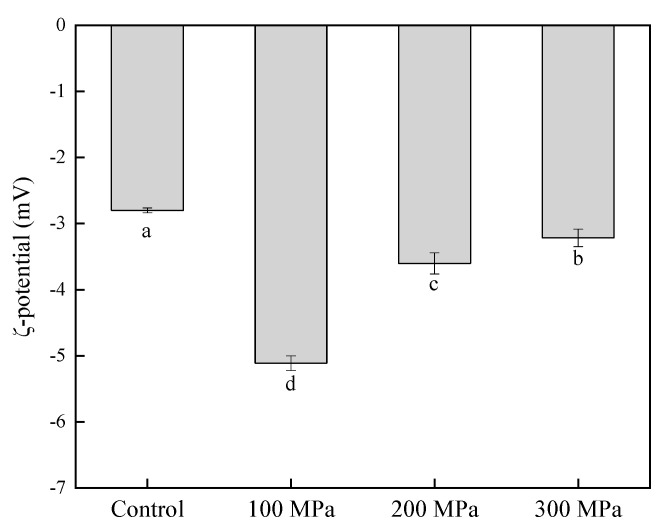
Effect of HHP pretreatment on the ζ-potential of RBPH. HHP: high hydrostatic pressure; RBPH: rice bran protein hydrolysates. Control, 100 MPa, 200 MPa, and 300 MPa represent RBPH samples subjected to 0, 100, 200, and 300 MPa pretreatment, respectively. Different lowercase letters indicate values that differ significantly (*p* < 0.05). All the HHP pretreatments were conducted at 25 °C for 30 min.

The existence of protein on the surface of the droplet may generate an electric charge [42]. ζ-potential can be used to determine the protein’s surface charge density, which provides a sign of the potential stability of an emulsion liquid system [43]. Figure 5 shows significant (*p* < 0.05) changes in the ζ-potential of the control and the RBPH samples pretreated by HHP of –2.81 mV (control group), –5.1 mV (100 MPa), –3.68 mV (200 MPa) and –3.25 mV (300 MPa), respectively.

Compared with the control group, the absolute value of RBPH’s ζ-potential in an aqueous dispersion was relatively larger. Theoretically, the absolute value of ζ-potential is associated with the colloidal stability of protein dispersion. In general, the absolute value of ζ-potential is positively correlated with intermolecular electrostatic repulsion. The greater the intermolecular repulsion, the better the stability of the colloid [44]. Therefore, RBPH pretreated by HHP had better colloidal stability. Compared with RBPH pretreated by HHP at 200 MPa and 300 MPa, RBPH pretreated at 100 MPa had a larger negative ζ-potential in the dispersion and showed better colloidal stability. The result was not in parallel to the result of Li et al. [19], who concluded that HHP treatment could not affect the volume of soybean protein isolate significantly, which results in the non-obvious electrostatic interaction change. Our result was different. It was possible that the raw materials of protein and processing methods were different. 

### 3.6. Effect of HHP Pretreatment on SDS-PAGE Profiles of RBPH

**Figure 6:**
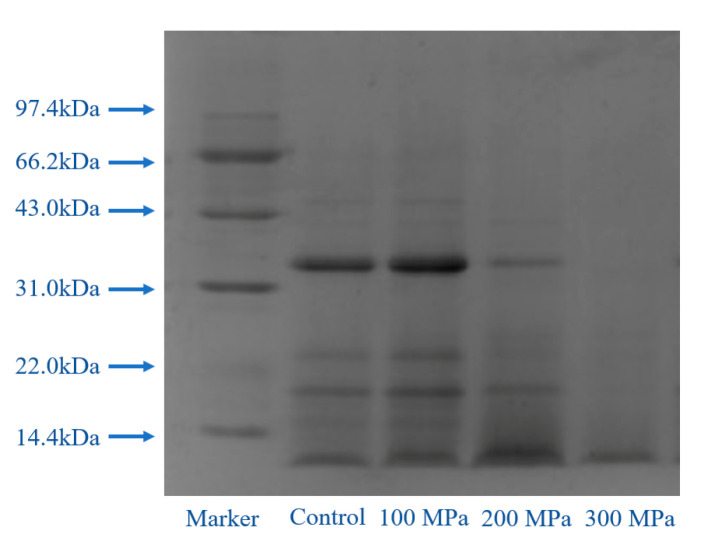
Effect of HHP pretreatment on the SDS-PAGE profiles of RBPH. HHP: high hydrostatic pressure; RBPH: rice bran protein hydrolysates; SDS-PAGE: sodium dodecyl sulfate-polyacrylamide gel electrophoresis. Control, 100 MPa, 200 MPa, and 300 MPa represent RBPH samples subjected to 0, 100, 200, and 300 MPa pretreatment, respectively. All the HHP pretreatments were conducted at 25 °C for 30 min.

The electrophoretic bands of the control group and RBPH treated at 100–300 MPa are shown in Figure 6. RBPH showed four high density protein bands, which were distributed between 31.0 kDa and 43.0 kDa, above 22.0 kDa, below 22.0 kDa, and below 14.4 kDa. This result was similar to the reports of Phongthai et al. [45] on RBPH’s composition. Compared with the control group and pretreatment at 100 MPa, the large subunit components of RBPH after HHP pretreatment at 200 MPa and 300 MPa disappeared, and the content of the small subunit components increased significantly, indicating that more macromolecular protein components were hydrolyzed into smaller components. As shown by the data in Figure 1 and Figure 4, the structure of RBP was unfolded after the HHP pretreatment (100 MPa, 200 MPa), which favored enzymatic hydrolysis. As the RBP molecules had a certain degree of dissociation and extension [20], RBP was easily hydrolyzed to a smaller particle size by trypsin. Thus, the solubility improved greatly. When the pressure was too high (300 MPa), the RBPH molecules aggregated, the particle size became larger, and the hidden hydrophobic groups were exposed [18]. The structural balance of the protein changed and its solubility decreased. However, the secondary bonds which caused protein molecule aggregation were destroyed under the SDS-PAGE conditions; thus, only the band below 14.4 kDa was found at 300 MPa.

Current studies on protein hydrolysis have indicated that HHP treatment can destroy the disulfide bond of protein [46], and that enzymatic hydrolysis can break the peptide bond, which can transform the protein into smaller peptide fragments [47]. A report has also shown that the enzymatic hydrolysis of ginkgo seeds treated with HHP was better than that under atmospheric pressure [29].

As reported, a protein’s structure can be changed by HHP, such as by unfolding, so that protease can enter the binding site for hydrolysis more effectively, increasing the sensitivity of protein to the enzyme [48]. With the increase in pressure, the bands of the RBPH subunits became lighter, which demonstrated that the RBPH structure had changed during the reaction. These findings are consistent with those of a previous study, which indicated that the structure of the protein changed after trypsin treatment [49]. Therefore, structure of RBPH pretreated by HHP was altered, resulting in changes in the functional properties.

### 3.7. Effect of HHP Pretreatment on the Free SH Group Content of RBPH

Free SH is a vital chemical bond that stabilizes the conformation of proteins [12]. It is usually used to characterize the changes in the structural and functional characteristics of proteins [50]. HHP pretreatment brought about some changes in SH content that appeared to be closely connected to protein unfolding. 

**Figure 7:**
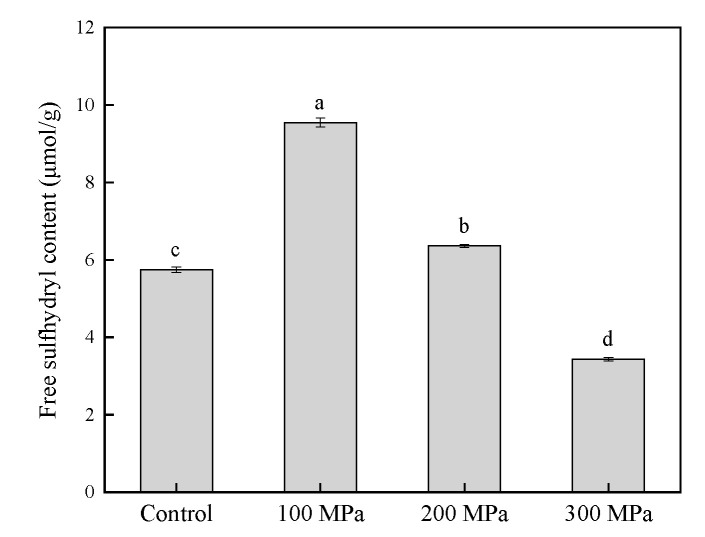
Effect of HHP pretreatment on the free sulfhydryl content of RBPH. HHP: high hydrostatic pressure; RBPH: rice bran protein hydrolysates. Control, 100 MPa, 200 MPa, and 300 MPa represent RBPH samples subjected to 0, 100, 200, and 300 MPa pretreatment, respectively. Different letters indicate values that differ significantly (*p* < 0.05). All the HHP pretreatments were conducted at 25 °C for 30 min.

Figure 7 shows that the SH content of RBPH samples was affected significantly by HHP pretreatment (*p* < 0.05); this result may be the reason why breaking of the noncovalent bonds induced unfolding of protein [51]. The SH content increased significantly (*p* < 0.05) at 100 MPa. However, when the pressure was higher than 100 MPa, the SH content of all RBPH samples gradually decreased as the pressure increased (*p* < 0.05). The result was similar to that of the SH content change trend found in a previous study [18]. The authors reported that HHP induced protein unfolding and subsequently aggregated the unfolded protein [18]. At 100 MPa, the free SH group may be stable, and the degree of aggregation was relatively low. However, under higher pressure, the protein disulfide bonds were re-formed prominently by a hydrophobic interaction which was caused by the aggregation of unfolded protein.

### 3.8. Effect of HHP Pretreatment on the Molecular Weight Distribution of RBPH

**Figure 8:**
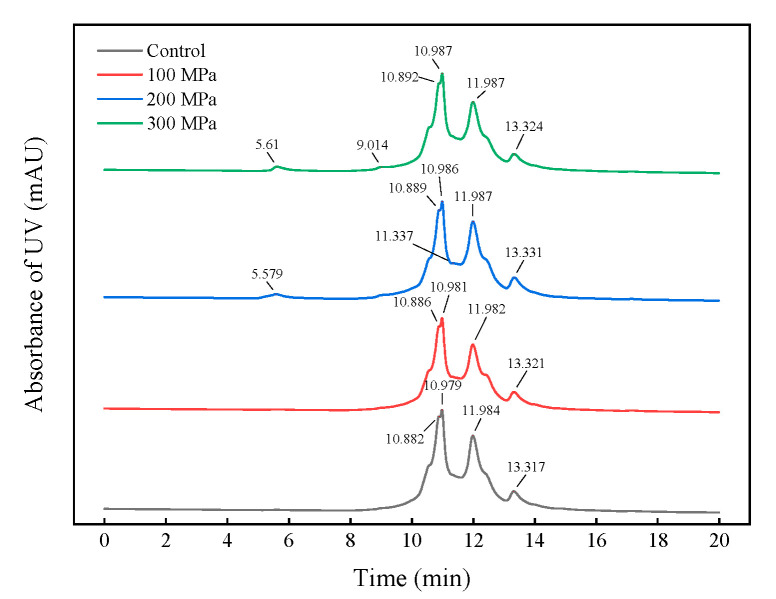
Effect of HHP pretreatment on the molecular-weight distribution profiles of RBPH. HHP: high hydrostatic pressure; RBPH: rice bran protein hydrolysates. Control, 100 MPa, 200 MPa, and 300 MPa represent RBPH samples subjected to 0, 100, 200, and 300 MPa pretreatment, respectively. All the HHP pretreatments were conducted at 25 °C for 30 min.

In this study, HPSEC was used to characterize the molecular weight distribution of RBPH pretreated by HHP. In the HPSEC profile of RBPH, there were four major elution peaks (Figure 8). These peaks were found at retention times of <10 min, 10–11 min, 11–12 min, and >13 min, which correspond to the molecular weight distribution above 27 kDa, 22–27 kDa, 18–22 kDa, and below 14 kDa, respectively (Table 1).

With the increase in pressure, the amount of RBPH with a molecular weight of 22–27 kDa decreased gradually, while the proportions of RBPH with a weight of 18–22 kDa and below 14 kDa increased. A small proportion of high-molecular-weight RBPH began to appear at 200 MPa. The amount of hydrolysate with a molecular weight above 27 kDa was greatest at 300 MPa out of all the samples. This phenomenon indicated that the hydrolysis and aggregation of protein occurred interactively. HHP pretreatment could unfold the protein structure to facilitate hydrolysis, and excessive pressure (300 MPa) pretreatment can lead to the aggregation of RBPH. This was the cause of the amount of low-molecular-weight RBPH (18–22 kDa, <14 kDa) reducing further at 300 MPa. The experiment data of this work (Figure 2) revealed that the emulsification of RBPH was optimal at 200 MPa, which indicated that peptide fragments of 18–22 kDa were positively correlated with emulsification. This finding was in accordance with the particle size results of this experiment (Figure 4). Similarly, Guan et al. [27] reported that a combined treatment with HHP and enzymes can change the molecular weight distribution of proteins.

### 3.9. Effect of HHP Pretreatment on the Surface Morphology of RBPH

**Figure 9:**
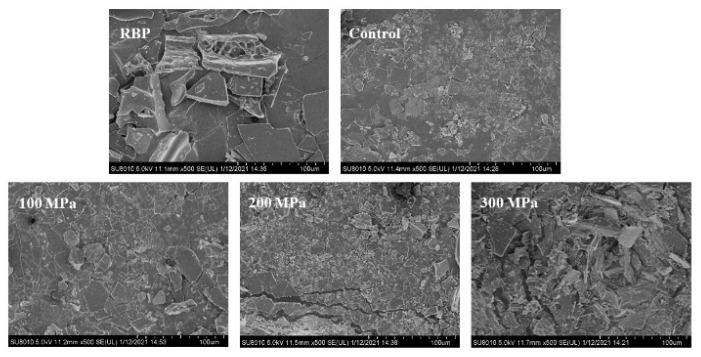
Effect of HHP pretreatment on the surface morphology of RBPH. HHP: high hydrostatic pressure; RBPH: rice bran protein hydrolysates; RBP: rice bran protein. Control, 100 MPa, 200 MPa, and 300 MPa represent RBPH samples subjected to 0, 100, 200, and 300 MPa pretreatment, respectively. All the HHP pretreatments were conducted at 25 °C for 30 min.

The surface morphology of RBP and RBPH is exhibited in Figure 9. The RBP sample shows a larger sheet structure with irregular edges. The samples hydrolyzed by trypsin comprised smaller particles with smooth edges. The structure of RBP was unfolded after HHP pretreatment, and hydrolysis treatment led to further disruption of the spatial stereoscopic network structure of RBP. RBPH particles showed a smaller and more uniform size with smoother edges at 100 MPa and 200 MPa. However, small particles decreased, and large particles increased when the HHP pretreatment was 300 MPa, which may be attributed to RBPH aggregation at higher pretreatment pressure. Generally, protein solubility can be affected by changes in particle size and structure [6]. The trend of the change in the morphology of RBPH was in accordance with the results seen for the solubility, emulsifying properties and particle size changes in this study (Figure 1, Figure 2 and Figure 4).

### 3.10. Effect of HHP Pretreatment on the FTIR Spectra of RBPH

**Figure 10:**
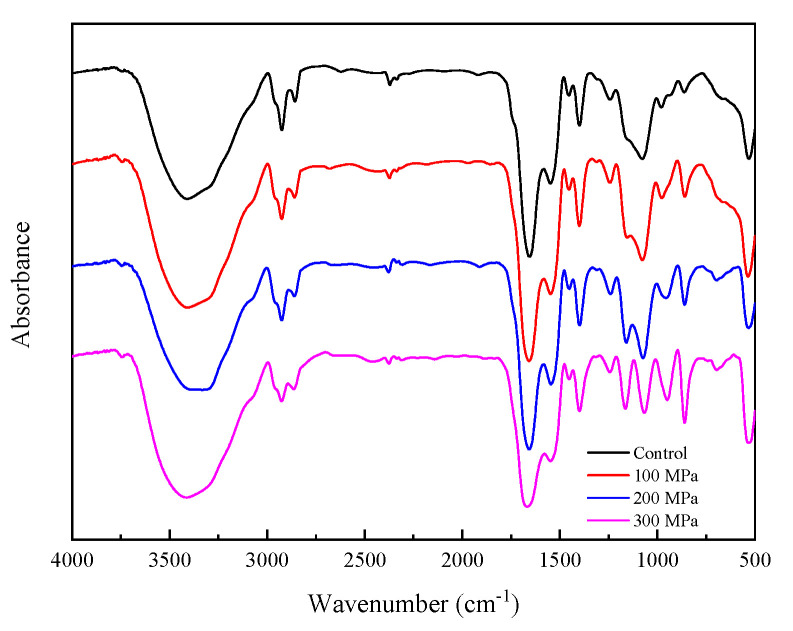
Effect of HHP pretreatment on the FTIR spectra of RBPH. HHP: high hydrostatic pressure; RBPH: rice bran protein hydrolysates; FTIR: Fourier transform infrared. Control, 100 MPa, 200 MPa, and 300 MPa represent RBPH samples subjected to 0, 100, 200, and 300 MPa pretreatment, respectively. All the HHP pretreatments were conducted at 25 °C for 30 min.

In this experiment, the secondary structure of RBPH was studied using FTIR spectroscopy. As shown in Figure 10, the infrared spectrum was from 500 cm^−1^ to 4000 cm^−1^. Changes in a protein’s secondary structure are generally evaluated by Amide I stretching (1700–1600 cm^−1^, C=O of the peptide bond) and the Amide II shift (1600–1500 cm^−1^, N–H bending and C–N stretching) [52,53,54,55]. It can be seen that some characteristic absorption peaks shifted after HHP pretreatment. The characteristic absorption peak of -OH stretching vibration in the control group was 3418 cm^−1^. When the pressure was 100 MPa, it shifted to 3409 cm^−1^. When the pressure increased, the characteristic peak continued to shift. The characteristic peak shifted to 3413 cm^−1^ at 300 MPa. The results showed that the characteristic absorption peak of -OH stretching vibration was a blue shift after HHP pretreatment, which may be due to the effect of the HHP pretreatment on the hydrogen bonds. The absorption peak at 2924 cm^−1^ represents the stretching vibration of -CH. It shifted to 2926 cm^−1^ after HHP pretreatment. And the stretching vibration peak of C=O shifted from 1654 cm^−1^ in the control group to 1667 cm^−1^ in the HHP pretreatment samples, and the characteristic peak of the stretching vibration of C-N shifted from 1545 cm^−1^ to 1547 cm^−1^. Generally speaking, a protein’s secondary structure is associated with the hydrogen bonds [56]. These results showed that, compared with the control group, RBPH’s secondary structure changed from ordered to disordered, and the environment of the functional groups changed, indicating that the conformation of RBPH changed after HHP pretreatment.

### 3.11. Effect of HHP Pretreatment on the Intrinsic Fluorescence Spectrum of RBPH

Generally, the environmental polarity of tryptophan (Trp) affects the emission fluorescence spectra of protein. Trp is a sensitive means of characterizing the conformation of proteins and is considered as a measure for estimating the changes in a protein’s tertiary structure [56]. A previous study had indicated that only when the maximum absorption wavelength (λ_max_) and fluorescence intensity change at the same time can the spatial structure of protein be characterized [57].

**Figure 11:**
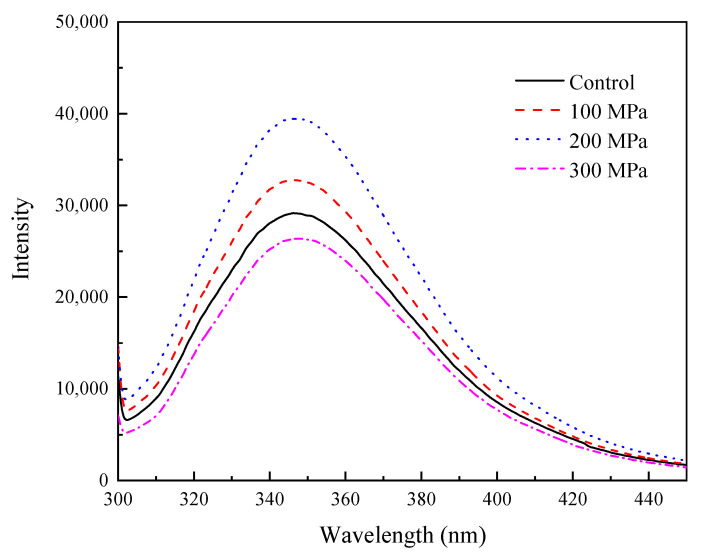
Effect of HHP pretreatment on the intrinsic fluorescence spectrum of RBPH. HHP: high hydrostatic pressure; RBPH: rice bran protein hydrolysates. Control, 100 MPa, 200 MPa, and 300 MPa represent RBPH samples subjected to 0, 100, 200, and 300 MPa pretreatment, respectively. All the HHP pretreatments were conducted at 25 °C for 30 min.

The intrinsic fluorescence spectrum of RBPH is depicted in Figure 11. As shown in this figure, the maximum emission wavelength and fluorescence intensity of RBPH were affected by different HHP pretreatment conditions. This result indicates that the relative fluorescence intensity of RBPH increased when the pressure was 100 MPa and 200 MPa, demonstrating that the hydrophobic groups were gradually exposed to the protein surface. With a further increase in pressure (300 MPa), the fluorescence intensity decreased to a level even lower than that of the control group, which indicated that the exposed hydrophobic groups might have undergone recombination or aggregation, making the protein structure steady [18]. At the same time, it was found that the maximum emission wavelength underwent a redshift with an increase in pressure, which indicated that HHP pretreatment increased the polarity of the environment around Trp, which may be the reason why the protein structure unfolded and the contact between Trp and water increased. The maximum emission wavelength of RBPH in the control group was 346 nm. The maximum emission wavelengths of RBPH treated with 100, 200, and 300 MPa were 347 nm, 348 nm, and 348 nm, respectively. This result was consistent with that of a previous study which reported that HHP could increase the relative fluorescence intensity at a pressure of 100 to 200 MPa, whereas it decreased at 300 to 400 MPa [20]. A fluorescence spectrum study confirmed that RBPH had undergone a conformational change through HHP pretreatment.

## 4. Conclusions

Overall, this work studied the structural and functional properties of RBPH pretreated by HHP. The current work indicated that RBPH exhibited significant improvements over the control in terms of the solubility, emulsifying properties, and foaming properties. The findings may indicate that HHP changed the spatial structure of RBP, thus enhancing the efficiency and effect of hydrolysis. The SDS-PAGE and free SH content results indicated that the primary structure of RBP altered after HHP pretreatment. The FTIR and fluorescence spectrum results showed that HHP changed the spatial structure of RBP. With the increase in pressure, the volume of the average particle size first decreased and then increased. The absolute value of the ζ-potential of the HHP-pretreated RBPH in an aqueous dispersion was larger than that of the control. Thus, the data obtained from the experiment indicated that HHP pretreatment could effectively improve the functionality of RBPH. This processing technology could broaden the potential application of RBP in food products. Further studies should be implemented to investigate the proper hydrolysis degree of RBP achieved through HHP for application in various food products.

## Figures and Tables

**Table 1:** Effect of HHP pretreatment on the molecular-weight distribution of RBPH.

Samples	Percentage Area of Peak (%) Corresponding to Retention Time/Molecular-Weight Distribution (min, kDa)			
	<10 min	10–11 min	11–12 min	>13 min
	>27 kDa	22–27 kDa	18–22 kDa	<14 kDa
Control	n.d.	51.66 ± 0.01 ^a^	39.61 ± 0.18 ^b^	8.73 ± 0.17 ^d^
100 MPa	n.d.	50.05 ± 0.11 ^c^	39.65 ± 0.04 ^b^	10.30 ± 0.07 ^a^
200 MPa	2.19 ± 0.02 ^b^	38.90 ± 0.11 ^d^	48.84 ± 0.06 ^a^	10.07 ± 0.03 ^b^
300 MPa	2.52 ± 0.01 ^a^	50.98 ± 0.12 ^b^	37.21 ± 0.03 ^c^	9.29 ± 0.08 ^c^

Control, 100 MPa, 200 MPa, and 300 MPa represent RBPH samples subjected to 0, 100, 200, and 300 MPa pretreatment, respectively. HHP: high hydrostatic pressure; RBPH: rice bran protein hydrolysates. The percentage area of peak represents the proportion of the integral of each peak area in the total integral of the peak areas. n.d., not detected. Different letters indicate values that differ significantly (*p* < 0.05). All the HHP pretreatments were conducted at 25 °C for 30 min.

## Data Availability

Not applicable.
